# One Single Site Clinical Study: To Evaluate the Safety and Efficacy of Immunotherapy With Autologous Dendritic Cells, Cytokine-Induced Killer Cells in Primary Hepatocellular Carcinoma Patients

**DOI:** 10.3389/fonc.2020.581270

**Published:** 2020-11-25

**Authors:** Kaiyue Xu, Zhengjie Meng, Xiaoxin Mu, Beicheng Sun, Yi Chai

**Affiliations:** ^1^ Department of Radiation Oncology, The First Affiliated Hospital of Nanjing Medical University, Nanjing, China; ^2^ College of Biotechnology and Pharmaceutical Engineering, Nanjing Tech University, Nanjing, China; ^3^ Hepatobiliary Center, The First Affiliated Hospital of Nanjing Medical University, Nanjing, China; ^4^ Key Laboratory of Liver Transplantation, Chinese Academy of Medical Sciences, Nanjing, China; ^5^ Department of Hepatobiliary Surgery, The Affiliated Drum Tower Hospital of Nanjing University Medical School, Nanjing, China

**Keywords:** dendritic cells, cytokine-induced killer cells, primary hepatocellular carcinoma, immunotherapy, CD24

## Abstract

Dendritic cells (DCs) and cytokine-induced killer (CIK) cells play an important role in the anti-tumor immune response. In this study, we evaluated the clinical effectiveness of DC/CIK-CD24 immunotherapies to primary hepatocellular carcinoma patients who received radical resection. 36 resected primary hepatocellular carcinoma (HCC) patients were enrolled from August 2014 to December 2015. All patients received two or four times of DC/CIK immunotherapy after radical resection. 1–4 years patients’ survival rates were evaluated during the follow-up. The 4-year survival rate of patients who received two times of immunotherapy was 47.1%, and the rate of those who received four times of immunotherapies was 52.6%. Compared to baseline, after receiving the DC/CIK-CD24 autotransfusion, the serum Treg concentration of the patients decreased, while CD3+, CD4+, CD56+ increased slightly. The adverse effect of immunotherapy was I–II° transient fever and could be tolerable. DC/CIK-CD24 immunotherapy can delay the relapse time.

## Introduction

Primary liver cancer (PHC) is one of the most common malignant tumors in China, and approximately 80% of liver cancer are hepatocellular carcinoma (HCC) (1). Surgery is effective in the early stage of liver cancer, but even in early stage hepatocellular carcinoma, surgical resection can’t solve the problem of recurrence of intrahepatic lesions or clearance of multiple lesions (1). Otherwise, only 5–10% of advanced HCC patients have the opportunity for resection. For patients with end-stage liver failure, there are problems with the lack of the donor and the use of immune escape (2). The only first-line drugs approved for unresectable patients with advanced hepatocellular carcinoma are sorafenib and regorafenib (3). The rate of patients’ response to sorafenib is low, and the drug resistance is not optimistic (4). Therefore, a new and effective therapy must be developed. In some international treatment guidelines (EASL and ASLD), immunotherapy is recommended for patients with advanced liver cancer (5). Studies on the immune mechanism of liver cancer have shown that the cellular immune function of the patients was significantly suppressed, and there is an extensive range of immune escape phenomenon (6). Therefore, inducing antigen-specific microenvironment immune response is a key point in the immunotherapy for liver cancer.

DC–CIK tumor autoimmune cell therapy technology refers to the mononuclear cells isolated from peripheral blood in patients, which are activated, amplified, and modified *in vitro* and then auto transfused into patients to regulate and enhance the immune function of the body; it directly kills tumor cells (4). The technology can produce two kinds of cells with different characteristics: the first kind of cells can medicate the immune response of patients, such as dendritic cells (DCs); the second kind of cells has inherent anti-tumor activity, such as cytokine-induced killer (CIK) cells.

Dendritic cells (DCs) are major antigen-presenting cells (APCs) that capture and present tumor antigens (7). Immature DCs stimulated by tumor antigen is activated to induce synthesis of cells and secretion of cytokines including interleukin-2 (IL-2), Tumor necrosis factor-α (TNF-α), interferon-β (IFN-β). These cytokines are able to activate tumor-specific immune response by stimulating the initial T cells (8).

Cytokine-induced killer (CIK) cells, a group of natural CD3+ CD56+ type II killer T lymphocytes (NKTs), are generated from the human peripheral blood mononuclear cells which can induce IFN-*γ*, anti-CD3 monoclonal antibodies, and IL-2 for a period of time (9, 10). CIK cells have the capacity of inhibiting proliferation and killing cancer cells.

The combination of DCs and CIK cells, the main cell types used in immunotherapy, enhances the immune response and kills tumor cells through their cytotoxic activity (7, 11, 12). Our previous results have proved that CD24 was a specific marker of liver cancer stem cells (13). Therefore, in this study, we loaded CD24 with DC–CIKs and explored the efficacy and safety of DC/CIK-CD24 cells’ reinfusion in HCC patients after radical resection of the tumor.

## Patients and Methods

In this study, we enrolled the patients who were diagnosed with primary hepatocellular carcinoma according to Chinese Clinical Guidelines which stand for grade I or II (14). CT or MRI excluded metastasis and liver cirrhosis at other sites. All patients had adequate bone marrow (leukocytes >4.0 × 10^9^/L; hemoglobin >120 g/L; platelets >100 × 10^9^/L) and renal functions. Child-Pugh score≦6, ECOG score 0–1. All patients were initially treated and had not received radiotherapy, chemotherapy, and immunotherapy before or after radical resection. From August 2014 to December 2015, 36 patients were enrolled in this study.

The mean age of the patients was 58.17 years old, and five of 36 were female. The primary endpoint of the study was survival rate for 1 to 4 years, the assessment of safety and quality of life.

### Treatments

Peripheral blood was collected from the patients on the 7th to 9th day after radical resection of the tumor. DC cells were injected subcutaneously 2 to 4 times per week, and CIK cells were injected subcutaneously 2 to 4 times every two days during the second week. Data collected included vital signs, ECOG, laboratory examination (whole blood cell count, liver and kidney function test, coagulation index, tumor biomarkers, immune biomarkers), DC/CIK-CD24 autologous transfusion information, toxicity, and adverse events. A safety visit was conducted 30 days after the last reinfusion. The patients were followed up every three months in the first year and every six months outpatient visit for 2 to 4 years.

### Preparation of DCs

50 ml autologous peripheral blood collected by hemocyte separator was transferred to a 50 ml centrifuge tube, 1,800r/min for 8 min, and discarded the plasma. Filled to original volume with saline solution, mixed well, and slowly added to the surface of Ficoll. Collected mononuclear cells by density gradient centrifugation were washed three times. Platelets were removed and supernatant were discarded, resuspended, and counted. The cell concentration was adjusted to 5 × 10^6^ cells/ml and incubated at 37°C in a 5% CO_2_ incubator for 30 min. DC culture was added (including 1% heat-inactivated autologous serum, 1,000 U/ml granulocyte-macrophage colony-stimulating factor, 500 units/ml IL-4)and incubated at 37° in 5% CO_2_ for seven days. Collected the DCs were washed three times with saline solution.

The harvested DCs were divided into four portions; one part was taken for patient infusion on the same day; one part was taken for quality control; the remaining DC cells were frozen in a −86°C refrigerator. On day 9, day 11, and day 13 they were injected into the patient once. The qualified DCs are not less than 1 × 10^7^ cells once.

### Peptide Loading

The CD24 peptide which was independently developed by our department with over 95% high-performance liquid chromatography-grade purity. The preparations were tested to be free of endotoxin. DCs were incubated with the CD24 peptide at a concentration of 10 μg/ml for 12 h on day 8. Harvested DCs were resuspended at a concentration of 1 × 10^6^/ml in 0.9% normal saline for intravenous infusion.

### Generation of CIKs

100 ml autologous peripheral blood collected by hemocyte separator was transferred to a 50 ml centrifuge tube, 1,800r/min for 8 min, and discarded the plasma. Filled to original volume with saline solution, mixed well, and slowly added to the surface of Ficoll. Mononuclear cells were collected by density gradient centrifugation and washed three times. Platelets were removed and the supernatant was discarded, resuspended, and counted. The cell concentration was adjusted to 1–2 × 10^6^ cells/ml and incubated at 37°C in a 5% CO_2_ incubator for 30 min. On the fourth day, 50ml CIK culture (including 1000 units/ml IFN-γ, after 24 h treatment, 50 ng/ml antibody against CD3, 1500 units/mL of IL2, and 1.5 ng/mL of IL-1α were added.) was added and incubated at 37°C, 5% CO_2_. On the sixth day, 140ml CIK culture was added. On the 7th day CIK culture was added to 1,000 ml and incubated at 37° in 5% CO_2_. The CIKs were collected and washed three times with saline solution. The supernatant was discarded and resuspended in 100 ml of normal saline; 1 g of human albumin was added into the cell suspension and mixed. The number of qualified CIK cells should be at least 2 × 10^9^ at a time.

### Statistical Analysis

Data were analyzed by using the SPSS16.0 software package (Chicago, IL, USA). Statistical significance was assessed through Paired-Sample *t* test. OS was the time between the first day of treatment and the date of death or the last day on which the patient was known to be alive. *P* values less than 0.05 (two-tailed) were considered significant.

## Results

### Characteristics of Immunotherapy

There were 17 patients who received DC/CIK-CD24 immunotherapy twice after radical resection of the tumor, and 19 patients received 4 times (**Table 1**). The average number of DC infusions was 8.6 ± 3.9 (10^6^), and the average number of CIK infusions was 15.8 ± 6.9 (10^8^; **Tables 2** and **3**).

**Table 1:** Patient characteristics.

Characteristic	Patients
**Number**	36
**Mean age**	58.17
**Gender**	
Male	31
Female	5
**ECOG performance status**	
0	20
1	16
**Stage status**	
Ia	9
II	27
**Frequency of immunotherapy**	
Two	17
Four	19
**HBV infection**	
Positive	17
Negative	19
**Recurrence/metastasis**	
Intrahepatic	25
Extrahepatic (lymph node, lung, brain)	11
**Tumor size (diameter)**	
>5 cm	18
<5 cm	19

**Table 2:** Average number of immune cells and adverse effects of patients who received immunotherapies twice (4 years follow-up).

Patient NO.	Sex	Size(cm × cm)	Average number of DC administrated (10^6^)	Average number of CIK administrated (10^8^)	4-year Metastasis site	Toxicity of skin	Non-infectivefever	1-year survival	2-year survival	3-year survival	4-year survival
01	M	4 × 4	8.8	17.1	liver	/	/				
02	M	2.5 × 3.7	8.6	15.8	/	/	/				
03	M	3.6 × 1.6	9.5	16.6	death	/	/				death
04	F	6.5 × 4.4	4.9	9.0	death	/	/	death			
05	M	4.5 × 3.1	8.1	15.1	liver	/	/				
06	M	5.5 × 3.4	11.0	19.7	death	/	I° transient fever				death
07	M	7.8 × 5.7	10.2	20.9	death	/	/		death		
08	M	3.5 × 3.3	7.8	13.5	lung	/	/				
09	M	6.7 × 4.4	8.9	14.1	death	/	/			death	
10	F	2.4 × 2.7	10.2	18.6	lymph node	/	/				
11	M	8.0 × 7.0	10.7	19.9	death	/	II° transient fever	death			
12	F	6.6 × 6.4	5.8	11.3	death	/	/		death		
13	M	3.6 × 2.7	9.4	16.6	/	/	/				
14	M	5.6 × 2.3	7.9	14.1	death	/	/	death			
15	M	1.7 × 1.6	8.6	15.3	/	/	/				
16	M	8.2 × 6.7	8.1	16.7	death	/	/				death
17	M	3.7 × 3.5	8.3	14.9	/	/	/				

**Table 3:** Average number of immune cells and adverse effects of patients who received immunotherapies 4 times (4 years follow up).

Patient NO.	Sex	Size(cm × cm)	Average number of DC administrated (10^6^)	Average number of CIK administrated (10^8^)	4-year Metastasis site	Toxicity of skin	Non-infective fever	1-year survival	2-year survival		3-year survival	4-year survival
01	F	3.1 × 2.6	17.3	32.7	/	/	/					
02	M	4.3 × 3.9	17.9	33.1	/	/	I° transient fever					
03	M	5.6 × 1.5	18.1	35.6	death	/	/		death			
04	M	2.5 × 2.1	17.4	34.8	/	/	/					
05	M	6.5 × 5.0	19.1	38.2	death	/	/		death			
06	M	6.5 × 4.4	18.4	33.2	liver	/	I° transient fever					
07	M	3.1 × 3.0	15.2	31.3	/	/	/					
08	M	4.5 × 3.4	16.9	34.8	death	/	/					death
09	M	10.7 × 6.4	12.9	25.3	death	/	/				death	
10	M	8.4 × 6.6	15.5	30.3	death	/	/	death				
11	F	5.0 × 3.0	17.5	36.1	liver	/	II° transient fever					
12	M	4.7 × 3.1	18.9	37.3	lymph node	/	/					
13	M	2.6 × 2.0	16.4	33.7	/	/	I° transient fever					
14	M	6.6 × 4.3	15.8	32.1	death	/	/		death			
15	M	3.7 × 2.6	17.9	35.5	/	/	II° transient fever					
16	M	4.2 × 2.0	14.3	28.5	death	/	/					death
17	M	6.6 × 4.5	16.7	33.5	death	/	/	death				
18	M	7.4 × 3.6	15.9	32.1	death	/	/	death				
19	M	5.3 × 4.0	16.1	32.2	lymph node	/	/					

### Adverse Effects

Of the 36 patients, transient fever was observed in 2 and 5 subjects who received 2 and 4 DC-CIK-CD24 infusions, respectively. The ratio was 11.8 and 26.3%, but the AE could be tolerable.

### Survival Rate

The clinical responses were assessed in two groups, respectively. All of the 36 included patients were assessed for OS. At the time of the analysis, there were 18 patients’ deaths, and 18 patients were confirmed alive. The 1-, 2-, 3-, and 4-year survival rate was 82.4, 70.6, 64.7, and 47.1% who received two times autotransfusion, respectively. The 1-, 2-, 3-, and 4-year survival rate of patients who received four times autotransfusion was 84.2, 68.4, 63.2, and 52.6%, respectively.

### Serum Immune Factors Concentration

We measured serum immune factors levels by ELISA after autotransfusion. In **Table 4** and **Table 5**, compared to baseline, the concentration of Treg, CD3+, CD4+, CD56+, CD19+ didn’t have significant change (P > 0.05), but in the group who received four times autotransfusion, the serum Treg concentration decreased while CD3+, CD4+, CD56+ increased slightly.

**Table 4:** Immune factors of patients who received DC/CIK-CD24 immunotherapies twice.

Immune factors	Baseline	After immunotherapies	*P* value
Treg	5.1 ± 1.0	5.4 ± 1.9	>0.05
PCD3+CD4+	65.1 ± 13.9	66.4 ± 9.3	>0.05
CD3+CD8+	38.9 ± 11.4	48.9 ± 13.6	>0.05
CD3+CD56+	20.7 ± 9.1	10.7 ± 4.3	>0.05
CD19	12.1 ± 3.9	10.1 ± 3.1	>0.05
CD4+/CD8+	1.2 ± 0.3	2.0 ± 0.5	>0.05

Data are means ± SD. Statistics of quantitative parameters are through Paired-Sample t test.

**Table 5:** Immune factors of patients who received DC/CIK-CD24 immunotherapies four times.

Immune factors	Baseline	After immunotherapies	*P* value
Treg	4.8 ± 1.1	4.2 ± 0.9	>0.05
CD3+CD4+	62.7 ± 9.3	68.4 ± 13.1	>0.05
CD3+CD8+	32.4 ± 9.6	42.3 ± 11.5	>0.05
CD3+CD56+	23.7 ± 4.1	24.3 ± 5.7	>0.05
CD19	14.5 ± 2.6	14.9 ± 3.5	>0.05
CD4+/CD8+	2.1 ± 0.1	1.7 ± 0.4	>0.05

Data are means ± SD. Statistics of quantitative parameters are through Paired-Sample t test.

## Discussion

As the most effective antigen-presenting cells, DCs have been applied as components of anti-tumor vaccines. DCs play an important role in anti-tumor immune response, such as capturing and presenting tumor-associated antigens and activating naive T cells, regulating T cell response, promoting cell synthesis, cytokine secretion **(** 15). Several studies have shown that the less number of DC infiltrates in the tumor, the worse is the prognosis of cancer patients (16). Therefore, DCs are essential in the initiation and maintenance of cellular immune response.

CIK cells, expression of CD3+CD56+, a subset of killer T lymphocytes, secrete cytokines including IFN-*γ* and TFN-α (16). With the help of DCs, the effective T cell stimulator, CIK cells can substantially enhance the effect of DC tumor vaccines (17). DC/CIK immunization therapy can generate effector CD8+ T cell infiltration and aggregation in the tumor region. The combination of DC and CIK may have potential therapeutic benefits in the long-term control of tumor progression. Because the HCC tumor is not sensitive to radiotherapy and chemotherapy, so in this study, we design the study to combine DC/CIK to immunize.

Because this is one single-site exploratory research, we didn’t recruit enough subjects, so we used historical comparison. According to clinical statistics, the 3-, 5-year survival rate of the primary HCC was 60.5 and 41.8%, respectively. Compared to the data, the survival rate of the subjects who received DC/CIK immunotherapy was slightly higher. In the groups who received four times autotransfusion, the serum Treg concentration decreased, while CD3+, CD4+, CD56+ increased; it seemed the DC/CIK immunotherapy had some effects on regulating body immune balance, but more information is needed to confirm.

Now, some studies have demonstrated that CD24 was overexpressed in hepatocellular carcinoma (HCC) tissues and the highly metastatic HCC cell lines (18 **,** 19). CD24 protected cancer cells escaped engulfing by macrophages. Our previous results also have shown that CD24 was an important biomarker of liver stem cells (13). Against CD24 is expected to become a new immunotherapy with a bright future. In this study, we loaded CD24 peptide with DC–CIKs, but because of the sample size, we didn’t detect the plasma CD24 level in HCC subjects.

In our study, we found the combination of DC/CIK-CD24 can improve the survival rates of liver cancer. It should be noted that in this study, the number of HCC subjects we enrolled was too limited, and the time of patients who received DC–CIK immunotherapy was not enough. Besides, it’s a single-site clinical trial, and the follow-up period was not long enough. Further rigorous multicentered, randomized clinical trials are required to confirm the effects of DC/CIK-CD24 immunotherapy in HCC.

## Data Availability Statement

The original contributions presented in the study are included in the article/supplementary material. Further inquiries can be directed to the corresponding author.

## Ethics Statement

The studies involving human participants were reviewed and approved by The First Affiliated Hospital of Nanjing Medical University. The patients/participants provided their written informed consent to participate in this study. Written informed consent was obtained from the individuals for the publication of any potentially identifiable images or data included in this article.

## Author Contributions

YC and XS provided the direction of this manuscript. KX, ZM and XM collected and analyzed the data. KX wrote the manuscript. YC revised this manuscript. All authors contributed to the article and approved the submitted version.

## Funding

This study was supported by National Science and Technology Major Project (2018ZX09734-007).

## Conflict of Interest

The authors declare that the research was conducted in the absence of any commercial or financial relationships that could be construed as a potential conflict of interest.
